# Non-invasive imaging of Toll-like receptor 5 expression using ^131^I-labeled mAb in the mice bearing H22 tumors

**DOI:** 10.3892/ol.2014.2025

**Published:** 2014-04-02

**Authors:** CHANGYA YANG, QINGYING YUN, HUKUI SUN, GUANGJIE YANG, TING LIANG, CHAO ZHANG, JING SONG, JIANKUI HAN, GUIHUA HOU

**Affiliations:** 1 Key Laboratory for Experimental Teratology of the Ministry of Education and Institute of Experimental Nuclear Medicine, School of Medicine, Shandong Univeristy, Jinan, Shandong 250012, P.R. China; 2 Department of Laboratory, The Second Affiliated Hospital, Shandong University of Traditional Chinese Medicine, Jinan, Shandong 250012, P.R. China; 3 Department of Nuclear Medicine, Qilu Hospital, School of Medicine, Shandong University, Jinan, Shandong 250012, P.R. China

**Keywords:** toll-like receptor 5, radioiodine, molecular imaging, autoradiography, hepatocellular carcinoma

## Abstract

Toll-like receptor 5 (TLR5) is overexpressed in several cancers and metastases, and presents an enticing target for molecular imaging of primary tumors. In the present study, ^131^I-anti-TLR5 monoclonal antibody (mAb) was evaluated for its use as a novel radiotracer for imaging hepatocarcinoma in mice bearing H22 tumors. The expression of TLR5 was analyzed by quantitative polymerase chain reaction and immunohistochemistry. The anti-TLR5 mAb and isotype immunoglobulin G (IgG) were radiolabeled with iodine-131 by the Iodogen method. The *in vitro* stability of iodinalized probes was determined in serum or saline for a series of times, and then evaluated with radio-thin-layer chromatography. The biodistribution study and autoradiography were performed in H22 tumor-bearing mice. It was found that H22-xenografted tumor tissue exhibited a higher level of TLR5 expression compared with normal liver tissues. ^131^I-anti-TLR5 mAb and ^131^I-IgG were obtained subsequent to purification, with high radiochemical purity (>95%), and remained stable for 48 h in human serum. The target-to-non-target ratio in the ^131^I-anti-TLR5 mAb group was significantly higher compared with the ^131^I-IgG group. The biodistribution study and autoradiography demonstrated that ^131^I-anti-TLR5 mAb was specifically retained in hepatocarcinoma with a high tumor uptake. Altogether, these results show that ^131^I-anti-TLR5 mAb is capable of detecting lesions in a TLR5-expressing tumor, with high target selectivity, and may offer a promising agent for hepatocarcinoma diagnosis and encourage further investigation.

## Introduction

Hepatocellular carcinoma (HCC) is the most common tumor that is highly aggressive and has a high recurrence (1). It is estimated that the majority of HCCs in China develop as a consequence of chronic infection with hepatitis B virus and arise in fibrotic or cirrhotic livers (2). However, early diagnosis of HCC remains a challenge, as the majority of patients have no symptoms in the early stage. Thus, there is a prominent requirement for oncology imaging modalities or biomarkers capable of identifying early-stage tumors, and signs of tumor progression and recurrence of HCC (3).

It is known that Toll-like receptors (TLRs) play prominent roles in inflammatory responses against pathogen infection. These receptors are primarily expressed on innate immune cells and recognize conserved pathogen-associated molecular patterns (4). TLR-expressing cells represent the first line of defense sensing pathogen invasion, triggering innate immune responses and subsequently initiating antigen-specific adaptive immunity. In addition to microbial molecules, TLRs can also recognize specific endogenous ligands, including heat shock proteins or fragments of extracellular matrix proteins (5,6). Current advancement in cancer immunobiology highlights these receptors as crucial actors involved in tumor growth and progression (7), while it was found that various TLRs exhibit either antitumor or protumor activities (8,9).

Previously, much attention has been paid to investigating the role of TLR5 in cancer progression and metastasis (10). Current studies show that TLR5 is expressed in multiple epithelial tissues, but also by several cancer cells. For example, the majority of human breast cancer samples also express TLR5 and there is an elevated expression of TLR5 in certain subtypes of breast carcinomas (11). It has also been shown that TLR5 is overexpressed in gastric carcinoma cells, and activation of TLR5 by flagellin provokes potent antitumor activity and thus inhibits the growth of colon tumors *in vivo* (12). By contrast, a study by Sfondrini *et al* (13) demonstrated that the early administration of flagellin simultaneous to implanting mouse mammary cells induced an increase in tumor growth. Currently, the particular function and exact mechanism of TLR5 signaling pathways in cancer cells remains poorly understood, and the abnormal expression of TLR5 has been noted as a potential biomarker for tumors. Therefore, TLR5 presents as an enticing target for molecular imaging of metastases and the metastatic potential of the primary tumor that expresses TLR5.

Due to its anatomical site, the liver is constantly exposed to gut-derived bacterial products, viral infection, alcohol or other products, which may be the reason for chronic liver damage, thus increasing the risk for HCC. Possibly as a consequence of this, TLRs play a key role in liver physiology and pathophysiology, due to their role in the immune system and their significant contribution to several biological processes, including promotion of epithelial regeneration and carcinogenesis (14). It has been demonstrated that there is a strong association between TLR3, TLR4 and TLR9 expression and tumor aggressiveness and poor prognosis in HCC (15). In addition, it was recently reported that the liver was a major target for TLR5 agonists and a key mediator of TLR5-dependent effects *in vivo* (16).

The main obstacle in the diagnosis of HCC is the low sensitivity for the detection of tumors <2 cm in size. The traditional imaging modalities indicated for small-HCC detection are contrast-enhanced ultrasound and contrast-enhanced magnetic resonance imaging (MRI) that have shown a high false-negative detection rate. Thus, a novel and more sensitive detection method is urgently required for the diagnosis of small HCC without a biopsy. Nuclear molecular imaging is such an emerging and promising science that has been applied in a broad range of clinical diagnoses and therapy. ^11^C-acetate and ^18^F-fluorodeoxyglucose (FDG) are complementary tracers in the role of a functional and biochemical probe for detecting both primary and secondary HCC through the degree of tumor cell differentiation. Although increasing evidence has shown that TLR5 plays a prominent role in cancer progression, its expression and role in HCC remain unclassified.

As aforementioned, we hypothesize that TLR5 may be a good biomarker for the detection of HCC, and therefore a radioiodinated anti-TRL5 monoclonal antibody (mAb) was prepared and its tumor-targeting potential was evaluated using the H22 hepatocarcinoma-bearing mice model.

## Materials and methods

### Cells and animals

The H22 hepatoma cell line was stored in our laboratory (Institute of Experimental Nuclear Medicine, School of Medicine, Shandong University, Shandong, China). The cells were cultured in Dulbecco’s modified Eagle’s medium (Gibco, Invitrogen Life Technologies, Grand Island, NY, USA) supplemented with 10% (v/v) fetal bovine serum (Gibco), 100 U/ml penicillin and 100 mg/l streptomycin (Beyotime Biotech, Ltd., Shanghai, China) in humidified air containing 5% CO_2_ at 37°C.

Female BALB/c mice, 6 and 8 weeks of age, were purchased from the Experimental Animal Center of Shandong University (Shangdong, China). The mice were inoculated subcutaneously on the rear flanks with 4×10^6^ H22 cells in 100 μl normal saline. The animals were used for biodistribution and autoradiography experiments when the tumor size reached 6–8 mm in diameter. All experimental protocols described in the present study were under the approval of the Ethics Review Committee for Animal Experimentation of Shandong University (Jinan, China).

### Semi-quantitative reverse transcription polymerase chain reaction (RT-PCR)

The TLR5 mRNA expression level in the H22 tumor-bearing mice was measured using RT-PCR. Briefly, total RNA was extracted in accordance with the manufacturer’s instructions and then reverse transcribed to cDNA using the Gene Amp RNA PCR kit in a DNA thermal cycler (Bio-Rad, Hercules, CA, USA). A non-template control was included in all experiments. Primer sequences were as follows: TLR5 forward, 5′-GCAGGATCATGGCATGTCAAC-3′ and reverse, 5′-AATGGTCAAGTTAGCATACTGGG-3′; GAPDH forward, 5′-AGGCCGGTGCTGAGTATGTC-3′ and reverse, 5′-TGCCTGCTTCACCACCTTCT-3′. The amplification products were separated on an agarose gel (Gene Ltd., Hong Kong, China) and visualized with ethidium bromide. The predicted size for TLR5 and GAPDH was 269 and 530 bp, respectively.

### Immunohistochemistry analysis

Sections (4 μm thick) cut from the archived paraffin blocks, were attached to slides and deparaffinized with toluene, and gradually dehydrated through a descending alcohol series. To block non-specific binding of the antibodies, the sections were incubated with 2% goat serum in phosphate-buffered saline (PBS; blocking buffer) for 2 h at room temperature. Subsequently, the slides were stained using the rabbit anti-mouse anti-TLR5 mAb (1:200; Santa Cruz Biotechnology, Inc., Santa Cruz, CA, USA) or immunoglobulin G (IgG; Beijing Biosynthesis Biotechnology Co., Ltd., Beijing, China) as the negative control. Positive expression was indicated by brownish-yellow granules in the plasma membrane of hepatoma cells for TLR5. The sections were analyzed using an Olympus DP72 digital camera (Olympus Co., Centre Valley, PA, USA) at a magnification of ×400, and images were captured.

### Radiopharmaceutical preparation

Sodium [^131^I] iodide was obtained from the China Institute of Atomic Energy (Beijing, China). Anti-TLR5 mAb and control IgG were labeled with ^131^I-NaI by the Iodogen method. Briefly, 100 μl 0.05 M phosphate buffer (PB) and 22.8 MBq ^131^I-NaI were added into the prepared Iodogen-coated tubes (Pierce Biotechnology Ltd., Rockford, IL, USA) and then 20 μg of anti-TLR5 mAb or IgG were added respectively. Subsequently, the mixture was incubated at room temperature for 15 min with occasional shaking. The reaction was quenched by incubation with 150 μl 0.05 M PB for 15 min at room temperature. Radiolabeled antibodies were then purified by size-exclusion chromatography using a PD-10 Sephadex G-25 column (GE-Healthcare, Diegem, Belgium). The radiochemical purity was measured by a Wipe Test/Well Counter (Caprac; Capintec, Inc., Ramsey, NJ, USA). The *in vitro* stability of the radiotracer was determined in a serial time in human serum (Fame Ltd., Beijing, China) or PBS (0.05 mol/l; pH 7.4) at 37°C, and analyzed by radio-thin-layer chromatography (TLC) Strip Scanner (Mini-Scan radio-TLC Strip Scanner; Bioscan, Inc., Washington, DC, USA).

### Whole-body autoradiography

The H22 tumor-bearing mice were injected via the tail vein with a PBS solution (100 μl) of ^131^I-anti-TLR5 mAb or ^131^I-IgG (0.74 MBq) respectively. To block the uptake in the thyroid gland, 5% potassium iodide was fed to the mice for three days before injection. Serial images were performed at 12, 24, 36 and 48 h post-injection. The anesthetized groups of mice (n=4, per group) were placed in the supine position on the storage phosphor screen plate for 15 min. Subsequently, the plate was scanned by the Cyclone Plus Storage Phosphor system (Perkin-Elmer, Waltham, MA, USA) and analyzed using the OptiQuant Acquisition software (Perkin-Elmer).

### Biodistribution studies

To validate the imaging studies and further quantify the ^131^I-mAb uptake, biodistribution studies were performed at various times in the H22 tumor-bearing mice model. The mice were administered 0.37 MBq of ^131^I-mAb (100 μl) via the lateral tail vein. Subsequently, groups of four mice were sacrificed by cervical dislocation at 12, 24, 36 and 48 h after injection, respectively. Blood was collected and the selected tissues were rapidly harvested, weighed and analyzed for total γ-counts by the Wipe Test/Well Counter. Data were corrected for radioactive decay and the radioactivity values were expressed as percentage of the injected dose [ID (%)] per organ, per gram of tissue and as T/NT (target/non-target) ratio.

### Data analysis

Data are expressed as the mean ± standard deviation and P<0.05 was considered to indicate a statistically significant difference. An unpaired two-tailed t-test was used, and statistical analysis was performed using PRIZM SPSS 15.0 software (SPSS, Inc., Chicago, IL, USA).

## Results

### Expression of TLR5 in H22 cell lines and tumor tissue

**Figure 1:**
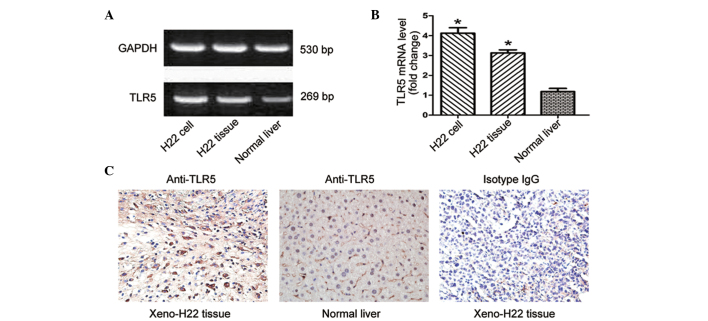
Analysis of TRL5 mRNA and protein expression. (A) mRNA expression and (B) relative mRNA expression of TLR5 by reverse transcription polymerase chain reaction in H22 cells, H22 xenograft tumor and normal mice liver tissue. ^*^P<0.05, vs. normal liver tissue. Each bar represents the mean±SD of three experiments. (C) Immunohistochemical staining of H22 xenograft tumor tissue and normal mice liver tissue with TLR5 mAb or isotype IgG. Magnification, ×400. Toll-like receptor 5; mAb, monoclonal antibody; IgG, immunoglobulin G.

The expression of TLR5 mRNA in the H22 cell line, H22 xenograft tumor tissue and normal liver tissue are shown in Fig. 1A. Compared with the normal liver group, the groups of the H22 cell line and H22 xenograft tumor tissue exhibited higher levels of TLR5 mRNA expression (P<0.05) (Fig. 1B). Consistent with the RT-PCR results, immunohistochemistry staining (Fig. 1C) showed that the expression of TLR5 was strongly localized among the tumor cells than the normal liver cells.

### Radiolabeling and stability assessment

**Figure 2:**
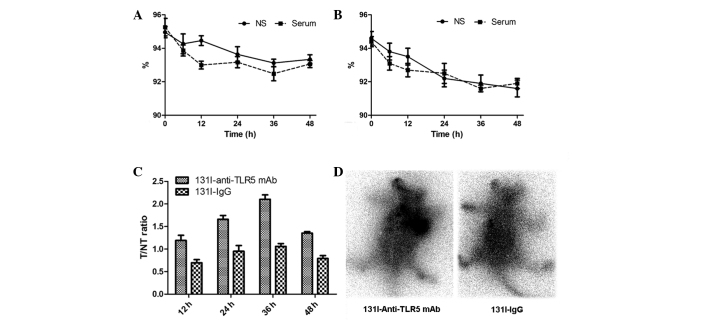
*In vitro* stability analysis of (A) ^131^I-anti-TLR5 mAb and (B) ^131^I-IgG are valued in serum or NS at various times. (C) The T/NT (target-to-non-target) ratios of ^131^I-IgG and ^131^I-anti-TLR5 mAb are defined as the tumor-to-liver percentage of the injected dose per gram of tissue ratio. Data are presented as a mean value±SD of four mice. (D) Representative whole-body autoradiography images of H22 tumor-bearing mice injected with ^131^I-anti-TLR5 mAb or ^131^I-IgG at 24 h following H22 cell injection. NS, no serum; TLR5, toll-like receptor 5; IgG, immunoglobulin G; mAb, monoclonal antibody.

^131^I-anti-TLR5 mAb and its control ^131^I-IgG were successfully radioiodinated. The radiochemical purity of ^131^I-anti-TLR5 mAb and ^131^I-IgG were both >95%. The specific activity of ^131^I-anti-TLR5 mAb and ^131^I-IgG was 29.56 and 25.43 GBq/μmol, respectively. ^131^I-anti-TLR5 mAb (Fig. 2A) and ^131^I-IgG (Fig. 2B) were stable *in vitro* for 48 h without an obvious decrease of radiochemical purity (>90%).

### Biodistribution studies

The tissue distributions of radioactivity at 12, 24, 36 and 48 h after injection are illustrated in Table I and II. During the first 12 h, a relatively high uptake of ^131^I-anti-TLR5 mAb was observed in the tumor site and in the blood, liver, kidney, spleen and lung. It is apparent that ^131^I-anti-TLR5 mAb localized at the site of the tumor to a significant extent at 24 h (ID/g reached ≤8.26±0.91%), and was retained there for >48 h (ID/g at 48 h: 2.17±0.53 %). However, there was no significant radioactivity in the tumor at all time points for ^131^I-IgG. The T/NT ratio is provided in Fig. 2C. It shows that the T/NT ratio for ^131^I-anti-TLR5 mAb was 1.19±0.20 at 12 h, and increased continually, eventually reaching ≤2.11±0.17 at 36 h. These ratios were significantly higher than that of the ^131^I-IgG group (P<0.05).

### Whole-body autoradiography

As shown in the imaging of autoradiography, it was found that ^131^I-anti-TLR5 became preferentially accumulated in the xenografted H22 tumor at 24 h (Fig. 2D), and then showed a gradual decline of uptake. Even 48 h after injection, H22 tumors were clearly visible in mice injected with ^131^I-anti-TLR5 mAb. While there was no clearly visualized accumulation in the tumor site in the group of ^131^I-IgG at all time points. Other organs with obvious uptake were the liver and kidneys. These results were consistent with the results detailed in Tables I and II, demonstrating that ^131^I-anti-TLR5 mAb appears to be more specifically retained in hepatocarcinoma than ^131^I-IgG.

## Discussion

Novel diagnostic imaging approaches for HCC have been developed during the past decades. Ultrasound scanning is non-invasive and widely used in clinical diagnosis of hepatoma, however the false-negative detection rate is >50% (17). Compared with the anatomical imaging strategies, including computed tomography and MRI, nuclear medicine imaging with radioisotope has the major advantage of high sensitivity. ^99m^Tc-methoxyisobutyl isonitrile and ^18^F-FDG have been commonly used for detection of HCC in the clinic (18), however, they are not specificity radiotracers for tumors. Therefore, solving the deficiency of specific-targeting probes in clinic is urgently required.

TLRs are extraordinarily notable in cancer research due to their role in a number of biological processes, including induction of innate and adaptive immune responses, carcinogenesis and regulation of inflammation. Previously, intense links have emerged between inflammation and the initiation and progression of several cancer types, including stomach, breast, ovary and liver (19). Chronic inflammation elicited by certain bacteria, for instance *Helicobacter pylori*, has been found to promote carcinogenesis (20). Activation of TLRs may favor a contribution for cancer progression and development, however, activation of various TLRs may exhibit contradictory results (21). It was reported that the activation of TLR4 signaling by lipopolysaccharide protects tumor cells from immune attack and therefore induces tumor growth (22,23). The activation of TLR9 on cancer cells could prevent apoptosis in cancer cells and stimulate proliferation of tumor cells (24). However, TLR3 exhibited an antiproliferative role in human breast cancer and melanoma (25). Thus, the function and biological significance of TLRs expressed on various tumor cells appears to be complicated.

Predominantly, TLRs may operate in two ways, which is dependent on the cell type. Cancer cells are more aggressive in response to TLRs activation, whilst immune cells usually respond to TLRs agonist by applying antitumor effects. Higher expression levels of TLR and the structural aberrations that characterize malignant epithelia, including the loss of cell polarity and abnormal intercellular junctions, may allow bacteria and their components to induce TLRs, therefore contributing to the disease progression (26).

As a pattern recognition receptor, TLR5 can recognize flagellin, which is a component of bacterial flagella. In malignant cells, TLR5 activates inflammatory responses and also induces invasion, migration and chemokine secretion (27). The significance of TLR5 in oral carcinoma has been demonstrated by assessing TLR5 expression in a cohort of 119 patients with oral tongue squamous cell carcinoma (28). Besides, TLR5 was identified as highly expressed and activated in breast carcinomas (11). Furthermore, activation of TLR5 by flagellin in breast cancer cells has been shown to alter the production of proinflammatory cytokines, and this creates potent antitumor activity in breast cancer, which may serve as a novel therapeutic target for human breast cancer therapy (29).

Therefore, the present study investigated the expression of TLR5 in the hepatocarcinoma cells. It was found that H22 cells and H22-xenografted tumor tissue exhibited higher levels of TLR5 expression than normal liver tissue, indicating that TLR5 may be a novel biomarker of hepatocarcinoma, although the mechanisms underlying remain far from understood.

The ^131^I-labeled anti-TLR5 mAb was also evaluated as a specific targeted radiotracer in H22 xenograft-bearing mice models. The biodistribution data showed that ^131^I-anti-TLR5 mAb had a high tumor uptake and T/NT ratio. In addition, the result of autoradiography showed that the radioactive accumulation in the tumor site became visible from 12 h post injection, and increased continually.

These results showed the potential of ^131^I-anti-TLR5 mAb as a promising molecular imaging agent for HCC diagnosis and encouraged further investigation. Nevertheless, since TLR5 mAb has a large molecular weight and an immunogenicity that may hinder its application in the clinic, it remains a great challenge to explore a novel small fragment of mAb or a small molecule with improved TLR5 targeting. In addition, to analyze the association between the expression of TLR5 in HCC and clinical stage, and to evaluate its significance in early-stage diagnosis, will be extremely helpful for the prognosis of patients suffering from HCC.

## Figures and Tables

**Table I:** Distribution of ^131^I-anti-TLR5-mAb in the H22 tumor-bearing mice.

Tissue	12 h	24 h	36 h	48 h
Blood	6.37±0.48	4.26±0.35	2.30±0.22	1.57±0.20
Heart	2.98±0.16	2.36±0.06	1.51±0.05	0.86±0.04
Liver	6.28±0.51	4.64±0.31	3.21±0.10	2.04±0.21
Spleen	2.83±0.14	2.95±0.17	1.39±0.04	1.22±0.06
Kidney	11.60±0.92	9.56±0.12	7.23±0.38	4.91±0.73
Stomach	1.70±0.27	1.04±0.06	0.86±0.03	0.41±0.02
Intestine	1.69±0.13	0.79±0.08	1.00±0.04	0.64±0.10
Bone	0.87±0.10	0.74±0.03	0.74±0.11	0.34±0.01
Muscle	0.98±0.05	0.99±0.03	0.48±0.07	0.23±0.02
Lung	2.79±0.40	1.69±0.10	1.08±0.14	0.94±0.08
Thyroid gland	1.62±0.04	1.24±0.07	0.77±0.04	0.58±0.01
Tumor	6.81±0.73	8.26±0.91	4.98±0.17	2.17±0.53

Data are presented as the mean±SD percentage of the injected dose per gram of tissue of four mice. TLR5, toll-like receptor 5; mAb, monoclonal antibody.

**Table II:** Distribution of ^131^I-IgG in the H22 tumor-bearing mice.

Tissue	12 h	24 h	36 h	48 h
Blood	6.16±0.43	3.69±0.12	2.09±0.07	1.12±0.24
Heart	3.43±0.19	2.01±0.09	1.40±0.25	0.42±0.08
Liver	5.79±0.41	4.15±0.07	3.73±0.13	1.03±0.08
Spleen	2.61±0.12	2.15±0.10	0.79±0.03	0.55±0.12
Kidney	10.51±1.08	8.20±0.80	5.38±0.33	3.71±0.29
Stomach	1.69±0.13	0.92±0.13	0.74±0.02	0.48±0.13
Intestine	1.48±0.25	1.02±0.09	0.87±0.14	0.45±0.11
Bone	1.17±0.19	0.95±0.27	1.02±0.06	0.36±0.05
Muscle	1.21±0.31	0.67±0.11	0.59±0.08	0.32±0.07
Lung	3.01±0.68	1.77±0.12	1.33±0.12	0.62±0.03
Thyroid gland	1.63±0.07	0.97±0.05	0.80±0.06	0.50±0.01
Tumor	3.83±0.26	3.27±0.34	2.68±0.06	1.13±0.18

Data are presented as the mean±SD percentage of the injected dose per gram of tissue of four mice. IgG, immunoglobulin G.
